# Introducing a novel mushroom from mycophagy community with emphasis on biomedical potency

**DOI:** 10.1371/journal.pone.0178050

**Published:** 2017-05-26

**Authors:** Somanjana Khatua, Arun Kumar Dutta, Swarnendu Chandra, Soumitra Paloi, Kanad Das, Krishnendu Acharya

**Affiliations:** 1 Molecular and Applied Mycology and Plant Pathology Laboratory, Centre of Advanced Study, Department of Botany, University of Calcutta, Kolkata, West Bengal, India; 2 Botanical Survey of India, Cryptogamic Unit, Howrah, India; Bharathidasan University, INDIA

## Abstract

Mushrooms have been prized by humankind as medicine and culinary wonder since antiquity. Though several species are ethnically valued; many prospective species are still being discovered. One such wild macrofungus has recently been discovered during subsequent field surveys in West Bengal, India which in turn exposed as a traditionally consumed popular myco-food. The collected taxon was found to be unique with regard to its morphological as well as genetical features. After detailed characterizations, the fungus was identified as a novel taxon belonging to the genus *Russula* (Russulaceae, Basidiomycota). Besides, the investigation was further extended in search of new functional ingredients and in this context, a water soluble crude polysaccharide rich extract (Rusalan) was isolated from dried basidiocarps. Accumulating evidences from GC-MS, HPTLC, FT-IR along with several spectrophotometric methods postulated that the fraction consisted mainly of carbohydrate in triple helical conformation, where glucose was the major monosaccharide mostly with β-type glycosidic linkage. Conversely, Rusalan showed pronounced antioxidant activity in six *in vitro* assay systems with EC_50_ value ranging from 190–1328 μg/ml concentration. The crude polysaccharide was also evaluated against six bacterial strains using microdilution method and the growth of *Staphylococcus aureus* and *Bacillus subtilis* were found to be inhibited effectively. In addition, immune-stimulatory assays demonstrated that Rusalan could evidently promote proliferation, induce phagocytosis, release NO, produce intracellular ROS and upregulate mRNA expression of iNOS, TNF-α, COX-2, as well as IL-6 genes in in mouse macrophage cells. Therefore, aim of the present study was not only to describe a new taxon to the world mycoflora but also to introduce a potent therapeutic agent that could be explored for food and pharmaceutical purposes. However, isolation of active component and *in vivo* studies need to be designed further.

## Introduction

Mushrooms have been appreciated since 3,000 BC due to their flavour, aroma and most importantly for medicinal metabolites. Consequently, they are long being used as dietary supplements for treatment and prevention of a multitude of diseases across the globe. This ethnically cultured health promoting concept has gradually matured over past decades through extensive scientific research for new and effective biomedicine [1]. Currently, investigators have isolated numerous active molecules from macrofungi and polysaccharides, especially β-glucan is considered as the most potent metabolite with diverse biological activities. These naturally occurring glucose polymers have been recognized as the best powerful immuno-stimulant and as a result fungal β-glucans are clinically used to activate the immune system function; particularly macrophages [2,3]. Macrophages act as the first line of defence against microbial infection, cancers and immunological diseases by initiating innate immune system followed by induction of adaptive immunity. They not only engulf foreign particulate matter, but also secrete several biological factors such as interleukin (IL) 6, IL 10, IL 1β, IL 12, transforming growth factor, (tumor necrosis factor) TNF-α and (nitric oxide) NO to invade foreign matters [4]. But when this macrophage-microbe interaction is excessively or inappropriately exploited application of agents able to improve host in born defence mechanism is highly recommended. Therefore, exhaustive research is now being directed in search of mushroom polysaccharides as effective immunomodulator [5,6]. Besides, there are previous report suggesting the high potential ability of β-glucans for inhibiting the growth of parasites, viruses and bacteria [7].

In recent years, significant increase of traditional antibiotics against antimicrobial infections has created an urgent health problem across the world. So, development of novel antimicrobial agents especially from natural sources can control infectious diseases along with decreasing emergence of new resistant strains [8]. In this regard, discovery of novel component that can inhibit growth of pathogenic microorganisms could be of considerable interest in search of products for therapeutic treatments.

Besides, mushroom polysaccharides have also emerged as an important source of antioxidants serving a critical role in protection of living organisms against free radical induced oxidative damage [9]. These radicals are known to be highly unstable and can readily react with surrounding molecules like protein, DNA, RNA, sugar and lipids. The reactions can lead to generate many severe disorders like cardiovascular, neurological, renal, liver, degenerative diseases and so forth [10]. Recently, there are several synthetic antioxidants available in market, though they are reported to execute several side effects including risk of carcinogenesis and liver damage. Thus, there is an urgent need for less toxic and more effective natural antioxidants [9]. In this regard, mushroom polysaccharides especially crude extracts appear to have the desired antioxidant activity [11].

Despite these therapeutic effects, research on macrofungi is not satisfactory as this diverse group still remains largely unexplored. To date, the number of mushrooms in world has been estimated approximately 150,000 of which only 10–15% have been scientifically described. It has also been postulated that to date, almost 60% of the novel species belong from tropics of which a large number are till to be described [12]. The Indian subcontinent is blessed with diverse agro-climatic zones harboring a treasure house of several mushrooms that in turn shaped indigenous knowledge. So far, approximately 850 mushroom species have been recorded only from India and more to discover yet [13]. In this context, our research group has been engaged to unveil the Indian myco-diversity mainly confining to the state, West Bengal and previously described three new species viz. *Russula kanadii* A.K. Dutta & K. Acharya [14], *Russula hookeri* S. Paloi, A.K. Dutta & K. Acharya [15] and *Russula buyckii* K. Acharya, S. Paloi & A.K. Dutta [16], all of those belong to one of the most abundant and widely distributed ectomycorrhizal agaric genera [17].

During repeated field surveys, one member of *Russula* was collected with the help of inhabited tribal communities during collection of food for their daily square meal. After detailed morphological and molecular characterizations, the species was confirmed as a novel taxon. Therefore, the present study was aimed for authentication of this new macrofungus with proper description and comparison with other related taxa. The work was further elaborated in order to contribute the mushroom towards its effective utilization. Accordingly, a crude polysaccharide rich extract was isolated from the novel species for analysing chemical composition and medicinal effects in order to estimate its immunomodulatory, antibacterial and antioxidant efficacy.

## Materials and methods

### Chemicals

2-Deoxy-D-ribose, ferric chloride, hydrogen peroxide, thiobarbituric acid, L-methionine, nitroblue tetrazolium (NBT), riboflavin, ferrozine, potassium ferricyanide, trichloroacetic acid, trifluoroacetic acid (TFA), 2, 2-Diphenyl-1-picrylhydrazyl (DPPH), sodium persulfate, ammonium molybdate, sodium borohydride, pyridine, toluene, dichloromethane, ascorbic acid, ethylene diaminetetraacetic acid (EDTA), butylated hydroxylanisole (BHA), gallic acid, bovine serum albumin (BSA), LPS (extraction from *Escherichia coli* 026:B6) and monosaccharides were procured from Sigma chemicals Co. (St. Louis, MO, USA). Toluene, DCM, and chloroform were of HPLC grade and monosaccharides were of extra pure form. A mushroom β glucan kit from Megazyme Institute Wicklow, Ireland was used. Dulbecco’s Modified Eagles Medium (DMEM), neutral red, sulfanilamide, naphthylethylenediamine dihydrochloride (NEDD), nutrient broth (NB), *p*-iodonitrotetrazolium chloride (INT), phosphoric acid, Congo red, 2´, 7´-Dichlorofluorescin diacetate (DCFDA), 4´, 6-Diamidino-2-Phenylindole (DAPI) were purchased from Himedia, Mumbai, India. WST and fetal bovine serum (FBS) were purchased from Takara Bio Inc, Japan and Invitrogen, Carlsbad, CA, USA respectively. PenStrep and amphotericin B were used from MP Biomedicals, Santa Ana, CA, USA.

### Morphological identification

Fresh basidiocarps of the specimen were collected during subsequent field trips (2011–2013) from West Bengal, India. The morphological and ecological features were noted in the field. The places from which the samples were collected were neither a conserved area nor any privately owned land. Thus, no specific permissions were required. Also, none of the field studies involved any endangered or protected species.

The collected basidiocarps were then taken to the laboratory and dried in a desiccator (40–50°C) for further preservation. The holotype specimen has been deposited in the Botanical Survey of India (CAL) and additional examined specimens in the Calcutta University Herbarium (CUH). Colour codes and terms (mostly) follow Methuen Handbook of Colour [18]. Microscopic features were performed following Dutta *et al*. [14] and Buyck [19]. Specimen was then examined with a Carl Zeiss AX10 Imager A1 phase contrast microscope. Q value denotes length/width ratio of the spores excluding ornamentation. Statistics for measurements of spores are based on 25 measurements from each of the four basidiocarps and given as a mean value (underlined); values in parentheses indicate minimum or maximum measured values. Scanning Electron Microscope (SEM) illustrations were obtained following Dutta *et al*. [14].

### Molecular identification

#### DNA extraction, PCR and cycle sequencing

Genomic DNA was extracted from dried herbarium specimen (10–50 mg) using the Fungal gDNA Mini Kit (Xcelris Genomics, Ahmedabad, India). PCR primers viz. ITS1 (5′ TCC GTA GGT GAA CCT GCG G 3′) and ITS4 (5′ TCC TCC GCT TAT TGA TAT GC 3′) were used for amplification of the nuclear ribosomal internal transcribed spacer (nrITS) region [20]. PCR cycling started with 4 min at 94°C, and was followed by 30 cycles consisting of denaturation for 30 s at 94°C, annealing for 30 s at 55°C, extension for 1 min at 72°C, and a final elongation step of 5 min at 72°C. PCR product was then observed using gel electrophoresis and purified using QIAquick® Gel Extraction Kit (QIAGEN, Germany). Cycle sequencing was performed with the help of ABI3730xl DNA Analyzer (Applied Biosystems, Carlsbad, California, USA) based on Sanger dideoxy sequencing technique using identical primers used for amplification of nrITS region. The newly generated sequences were deposited in GenBank (www.ncbi.nlm.nih.gov).

#### Dataset representation

The dataset representing the nrITS regions were created consisting of original sequence plus sequences acquired from Genbank. Twenty-eight nrITS sequences representing 16 species were used in the analyses, of which 24 were retrieved from GenBank and 4 sequences were generated for this study. Twenty-six sequences in the analysis represent genera *Russula*, including respective types for the subsect. *Auratinae* (*R*. *aurea* Pers.), *Melliolentinae* (*R*. *melliolens* Quél.), *Xerampelinae* (*R*. *xerampelina* (Schaeff.) Fr.) and *Paraintegrinae* (*R*. *integriformis* Sarnari). *Stereum hirsutum* (Willd.) Pers. and *Amylostereum laevigatum* (Fr.) Boidin were used for rooting purposes following Buyck *et al*. [17].

#### Sequence alignment and phylogenetic analysis

The nrITS sequences were edited with the CodonCode Aligner software (CodonCode Corporation, Dedham, Massachusetts). The edited sequences were then used for BLAST searches in the GenBank database. The newly generated nrITS, along with sequences retrieved from the GenBank based on a BLAST search were aligned using ClustalX [21] with default setting. Both the alignments were then imported into MEGA v. 6.0 [22] for additional manual adjustments.

Bayesian phylogenetic analyses (BA) were carried out using MrBayes v. 3.2.2 [23]. This program performs a Bayesian Inference (BI) of the phylogeny, using Metro-polis-coupled Markov chain Monte Carlo analyses [24]. For a given data set, the General time reversible (GTR) model was employed with gamma-distributed substitution rates. Markov chains were run for 10^6^ generations, saving a tree every 100^th^ generation. Default settings in MrBayes were used for the incremental heating scheme for the chains (1 cold and 3 heated chain), unconstrained branch length, and uninformative topology (uniform) priors. In each analysis, initial sampling trees (25%) were excluded as the burn-in phase. MrBayes was used to compute a 50% majority rule consensus of the remaining trees to obtain estimates of the Bayesian posterior probabilities (PPs) of the groups. PP values greater than 0.50 are indicated in the resulting trees.

#### Nomenclature

The electronic version of this article in Portable Document Format (PDF) in a work with an ISSN or ISBN will represent a published work according to the International Code of Nomenclature for algae, fungi, and plants, and hence the new names contained in the electronic publication of a PLOS ONE article are effectively published under that Code from the electronic edition alone, so there is no longer any need to provide printed copies. The new taxon described in this work has been submitted to MycoBank from where they will be made available to the Global Names Index. The unique MycoBank number can be resolved and the associated information viewed through any standard web browser by appending the MycoBank number contained in this publication to the prefix http://www.mycobank.org/MB/. The online version of this work is archived and available from the following digital repositories: PubMed Central, LOCKSS.

### Extraction of crude polysaccharide (Rusalan)

The extraction procedure was followed according to Palacios *et al*. [25] with modifications. Dried and powdered fruit bodies were steeped with ethanol (approximately 10 volume) for 2 days to eliminate the alcohol soluble components and filtered residue was re-extracted with ethanol. The air-dried filtrate was suspended and refluxed with distilled water at boiling condition for 7 h. Subsequently the extract was filtered through nylon cloth and lyophilised to concentrate. Four volumes of absolute ethanol were added for harvesting polysaccharide and incubated overnight at 4°C. After centrifugation, pellets were dissolved in water repeatedly to obtain crude polysaccharide fraction that is highly soluble in water. Alcohol precipitated pellets were recovered by centrifugation followed by washing with ethanol and acetone. The crude polysaccharide fraction was designated as Rusalan and kept in amber containers under dry condition until analysed.

### Structural characterization of Rusalan

#### Determination of total carbohydrate, glucan and protein

Total sugar content was measured by phenol sulphuric acid method using glucose as standard [26] and results were expressed as gm of glucose equivalents/100 gm of the dry polysaccharide. Total glucan, α-Glucan and β-glucan of the crude polysaccharide were calculated using mushroom and yeast β-glucan assay kit as per the protocol. All values of glucan contained were expressed as gm of glucose equivalents/100 gm of crude dry polysaccharide. Protein concentration was determined using the method described by Bradford [27]. Total protein content of the fraction was expressed as gm of BSA equivalents/100 gm of dry polysaccharide.

#### Depiction of helical conformation by congo red reaction

The helical structure of Rusalan was analysed by characterizing Congo red-polysaccharide reaction according to the method described by He *et al*. [28]. Different sets of solutions were prepared containing polysaccharide (0.5 mg/ml) in 0–0.5 M NaOH (increasing stepwise by 0.05 M increments) and 91 μM of Congo red. These were analysed in the range of 400–700 nm and the maximum absorption wavelengths were recorded as a function of NaOH concentration.

#### Fourier transform infrared spectroscopy (FT-IR)

The FT-IR spectra were recorded on PerkinElmer Precisely Spectrum 100 Model (USA). The crude polysaccharides were powdered with potassium bromide and pressed forming pellets for FT-IR detection in frequency range 400–4000 cm^-1^.

#### Determination of monosaccharide composition

10 mg Rusalan was hydrolysed with 2M TFA (5 ml) at 100°C for 2 h in a screw cap vial. TFA was removed by evaporation at 55°C under reduced pressure (Rotavapor R3, Butchi, Switzerland). The hydrolysate was dissolved by adding 1 ml of 50% ethanol followed by centrifugation (12,000 rpm, 5 min) to remove non-hydrolysed polysaccharide and subjected for monosaccharide composition analysis by HPTLC and GC-MS as described in our previous publication [29].

### Evaluation of antioxidant activities of Rusalan *in vitro*

The assay of total antioxidant capacity was carried out as described by Prieto, Pineda and Aguilar [30] and activity was expressed as number of equivalents of ascorbic acid. Besides, superoxide radical scavenging activity of Rusalan (100–1000 μg/ml) was evaluated using riboflavin-light-NBT system based on the method of Martinez *et al*. [31]. The technique explained by Halliwell, Gutteridge and Arumo [32] was followed for determination of hydroxyl radical scavenging activity. These free radicals were produced by Fenton’s reaction in existence of variable concentrations (500–1500 μg/ml) of Rusalan and absorbance was measured at 535 nm. In addition, the antioxidant activity was also evaluated using DPPH radical based on assay described by Ekanayake *et al*. [33]. DMSO solution of the radical (0.1 mM) was evaluated against various concentrations of crude polysaccharide (1000–2000 μg/ml) and absorbance was detected at 517 nm. Moreover, the ability of investigated extract to chelate ferrous ion was determined [34] using different concentrations of Rusalan (100–300 μg/ml). A modified method of reducing power as described by Oyaizu [35] was also considered. Variable doses of Rusalan (500–1500 μg/ml) were mixed in 1.5 ml reaction mixture and the absorbance was measured at 700 nm. Ascorbic acid was used for comparison in reducing power and total antioxidant capacity techniques. BHA was considered as standard in superoxide, DPPH and hydroxyl radical scavenging assays; while EDTA was adopted as a positive control in chelating ability of ferrous ion method. The sample concentrations providing 0.5 of absorbance or 50% of antioxidant activity were calculated from graphs of antioxidant activity percentages and regarded as EC_50_ value.

### Estimation of antibacterial potentiality of Rusalan

*Bacillus subtilis* MTCC Code 736, *Listeria monocytogenes* MTCC Code 657, *Staphylococcus aureus* MTCC Code 96, *Escherichia coli* MTCC Code 68, *Klebsiella pneumoniae* MTCC Code 109 and *Salmonella typhimurium* MTCC Code 98 were obtained from MTCC, Institute of Microbial Technology, Chandigarh, India. Antibacterial effect was estimated by determining minimum inhibitory concentration (MIC) values according to microdilution method [36]. The six investigating bacteria were cultured freshly and 1×10^5^ CFU/ml concentrated dilutions were prepared separately. Reactions were performed in 96 well plate consisting of 200 μl of NB, 20 μl of inoculum and different dilutions of Rusalan. Following incubation for 24 h at 37°C, 40 μl of INT dye (0.2 mg/ml) was added to each well and incubated for another 30 min. Concentration that inhibited 50% growth of bacteria in comparison with positive control was calculated as MIC value. Streptomycin was used as a standard drug.

### Determination of immuno-modulatory activity of Rusalan

#### Cell culture

RAW 264.7 mouse macrophage cells were purchased from NCCS, Pune, India. The cell line was maintained in DMEM supplemented with 10% (v/v) FBS, 0.5% (v/v) PenStrep (5,000 IU/ml penicillin and 5 mg/ml streptomycin) and 0.25% (v/v) amphotericin B (250 µg/ml). Cell line was maintained at 37°C in a humidified atmosphere with 5% CO_2_. In all experimental sets, LPS at 5 µg/ml concentration was used as a positive control. The studies were carried out on commercially available murine cell line; thus, no ethical clearance applies in this case.

#### Effect on Raw 264.7 cell proliferation

Increase in cell proliferation was determined using WST assay. Briefly, about 3000 cells were seeded in a 96-well plate overnight, Rusalan (50, 100, 200 and 400 µg/ml) was added to the 200 µl reaction mixture and incubated for different time intervals at 37°C. 20 µl WST reagent was added after 24h as well as 48 h treatment and absorbance was measured using a spectrophotometer at a wavelength of 450 nm.

#### Effect on phagocytic activity of macrophage cells

3000 cells/well were plated in 96 well plate overnight and then exposed to serial concentrations of Rusalan (50, 100, 20, 400 μg/ml) as the experimental group in 200 μl reaction mixture for 24 and 48 h. After treatment, reaction mixture was discarded and incubated with 100 μl DMEM media containing 0.07% (w/v) neutral red for 1 h followed by washing the cells with PBS twice. Then 100 μl cell lysate solution (ethanol and 0.01% acetic acid at the ratio of 1:1) was added into each well to lyse cells at room temperature for 2 h. The optical density at 575 nm was measured by a microplate reader [37].

#### Effect on NO production in macrophages

3000 cells/well were seeded in 96 well microplate overnight and then exposed to serial concentration of Rusalan (50, 100, 200, 400 μg/ml) as the experimental group in 200 μl reaction mixture for 24 h. After incubation, 100 μl of culture supernatant was mixed with an equal volume of the Griess reagent (1% sulfanilamide, 0.1% naphthylethylenediamine dihydrochloride, and 5% H_3_PO_4_). The mixture was incubated at room temperature for 10 min and absorbance was measured at 545 nm. The nitrite concentration was determined by extrapolation based on sodium nitrite (1–200 μM in culture medium) curve [38].

#### Detection of morphological changes of macrophages

Raw cells (1 × 10^4^ cells/well) were treated with various concentrations of Rusalan (50, 100, 200 and 400 µg/ml) and allowed for incubation of 24 h. The adherent cells were fixed with methanol for 10 min, washed with PBS and incubated with DAPI solution (1 µg/ml in PBS) for 15 min in dark. Excess dye was removed by washing cells three times with PBS. Cellular morphology was viewed and photographed using fluorescent microscope (FLoid Cell Imaging Station, Life Technologies, India).

#### Effect on production of intracellular reactive oxygen species (ROS)

RAW 264.7 cells were seeded (6 × 10^5^ cells/well) in 6 well plates and treated with increasing concentrations of Rusalan (50, 100, 200, 400 μg/ml) for 24 h. After treatment, cells were harvested and incubated with ROS indicator, DCFDA, to a final concentration of 5 μM in dark at 37°C for 30 min [39]. The intracellular ROS levels were measured with flow cytometry (BD Bioscience, USA) and analysed by BD CellQuest Pro software.

### Measurement of gene expression by reverse transcriptase-PCR analysis

Raw cells (6 × 10^5^ cells/well) in 6 well plates were treated with various concentrations of Rusalan (50, 100 and 200 µg/ml) and allowed for incubation of 24 h. RNA was extracted from cultured cells using TRIzol reagent and reverse transcription was carried out with 1 µg of total RNA using RT-and GO Mastermix. The reaction was carried out at 42 ºC for 60 min followed by 70 ºC for 10 min. COX-2, IL 6, iNOS, TNF-α genes were co-amplified with β-actin gene as control. The sequences of PCR primers are listed in Table 1. The PCR cycle conditions were as follows: 95 ºC for 4 min, then 35 cycles of 94 ºC for 20 s, annealing temperature (Tm) for specific primers for 30 s and 72 ºC for 45 s with a final extension step of 7 min at 72 ºC. The PCR products were separated in agarose gel, visualized under UV transilluminator and then photographed. For each gel, ImageJ software (http://rsbweb.nih.gov/ij/index.html) was applied for quantitative estimation of band intensity.

**Table 1:** Sequence of primers.

Sl No.	Primer Name	Primer Sequence	Tm (°C)	Reference
1	iNOS	F5′ GAGCGAGTTGTGGATTGTC 3′	55	[40]
		R5′ GGGAGGAGCTGATGGAGT 3′		
2	COX-2	F5′ CCCCCACAGTCAAAGACACT 3′	57	[41]
		R5′ GAGTCCATGTTCCAGGAGGA 3′		
3	IL-6	F5′ TTCCTCTCTGCAAGAGACT 3′	51	[41]
		R5′ TGTATCTCTCTGAAGGACT 3′		
4	TNF-α	F5′ ATGAGCACAGAAAGCATGATC 3′	56	[41]
		R5′ TACAGGCTTGTCACTCGAATT 3′		
5	β-Actin	F5′ GCTGTCCCTGTATGCCTCT 3′	55	[40]
		R5′ TTGATGTCACGCACGATTT 3′		

### Statistical analysis

All data are presented herein as mean ± standard deviation of three independent experiments each in triplicate. Calculations were performed using statistical package for Microsoft^®^ Office Excel (Microsoft^®^, USA) and differences were evaluated by means of one-way analysis of variance (ANOVA).

## Results and discussion

### Taxonomy

*Russula alatoreticula* K. Acharya, S. Khatua, A.K. Dutta & S. Paloi, sp. nov.

**MycoBank.** MB 814640.

**Etymology.** ‘alatoreticula’ is the Latin transliteration of English, winged little nets, for the typical reticulate-winged ornamentation of the basidiospore.

**Holotype.** India, West Bengal, West Midnapur district, Gurguripal, 22°25'49.13" N, 87°12'57.26" E, alt. 45 m, S. Paloi, 22 August 2013, CAL-1271.

#### Diagnostic description

Basidiomycota: Russulales: Russulaceae. Medium-sized (40–50 mm), plano-convex to finally infundibuliform, pastel red to dull red or greyish red pileus that turns reddish-orange with KOH; white lamellae turning yellowish-orange with KOH, absence of lamellulae; entirely white stipe; white spore print; mild taste; globose to subglobose (6.5–7.5 × 5.4–7.2 μm; Q = 1.1) basidiospores, with nearly complete reticulum to reticulate-winged ornamentation, amyloid suprahilar spot; presence of hymenial cystidia shaped clavate to subclavate with capitates to moniliform apex on gill sides (60–68 × 10.5–11.5 μm) and edges (43–50 × 10–11 μm); and presence of narrow (3–4 μm) caulocystidia.

**Fig 1:**
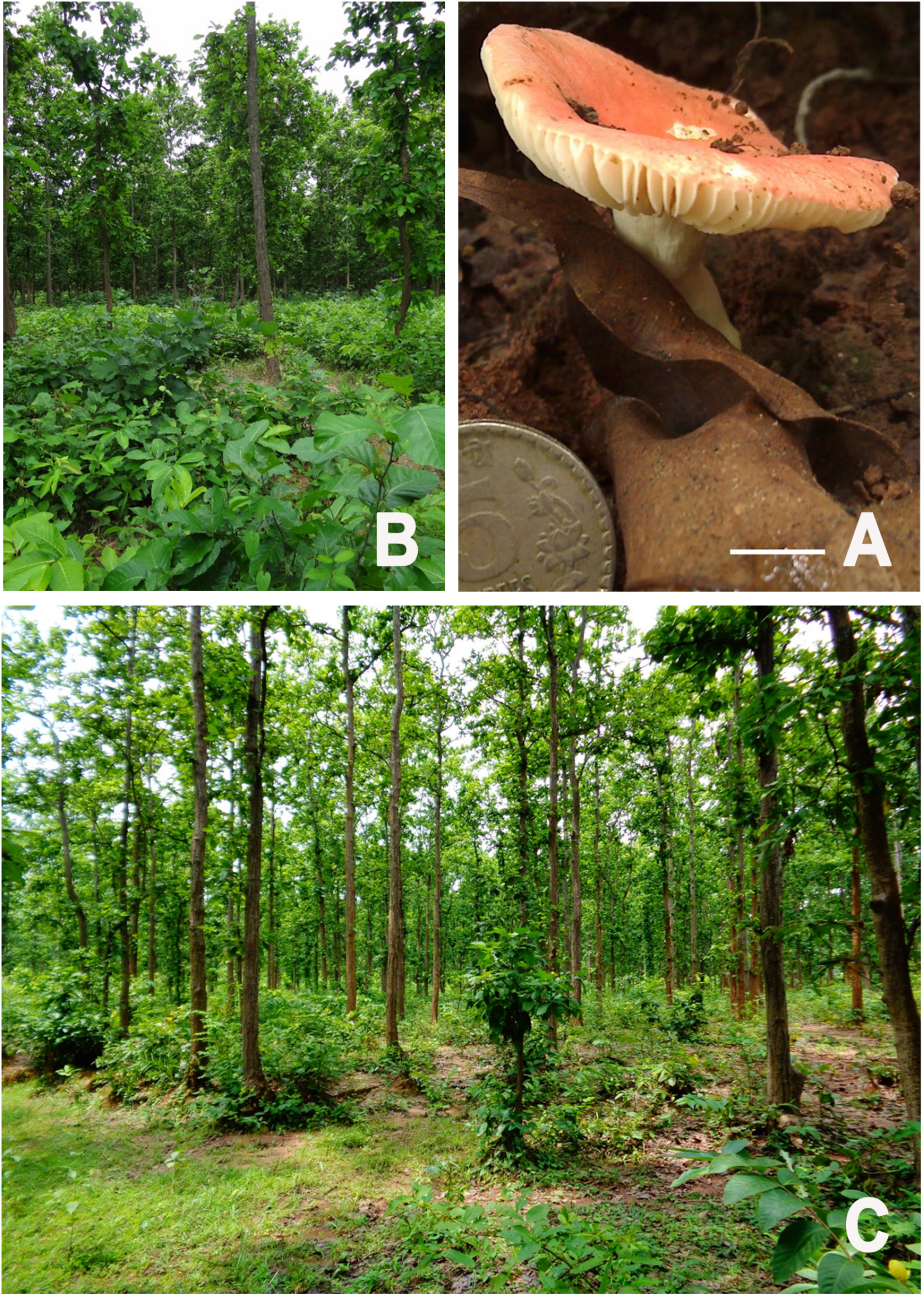
*Russula alatoreticula* (CAL-1271). (A) Fresh Basidiomata in the field. (B-C) Habitat—*Shorea robusta* dominated areas of Gurguripal and Khairulachak, West Bengal from where basidiomata of *Russula alatoreticula* were collected (photographs by S. Paloi). Scale bars: 10 mm in A.

Pileus 40–50 mm diam., at first convex, becoming plano-convex with a depressed centre, then infundibuliform with maturity (Fig 1A); surface smooth, pastel red (9A5) to dull red (10B4) or greyish red (10B5), often rose pale red (11A4), centre red (9A6-7; 9B7) to reddish brown (9D7-8), no colour change on exposure or with NH_4_OH, turns reddish-orange orange with KOH, scarlet red with FeSO_4_; margin reflexed at maturity, moderately striate towards margin; context 2 mm thick, white, translucent with NH_4_OH, turns yellowish-orange to 9H with KOH and fulvous with FeSO_4_. Lamellae adnexed, 4 mm broad, subdistatnt (spacing ca. 1 mm at pileus margin) after maturity, regular, white (1A1), unchanging after bruising, turning yellowish-orange with KOH, fulvous with FeSO_4_ and orange with sulphovanillin; edge concolorous; lamellulae absent. Stipe 31 × 10 mm, central, straight, cylindric with tapered at base, smooth, white (1A1), moist. Context solid in stipe, white (1A1), unchanging after bruising, apricot with FeSO_4_ which later turns into light snuff brown, no colour change with guaiac and phenol, orangish with KOH. Taste mild. Odour indistinctive. Spore print white.

**Fig 2:**
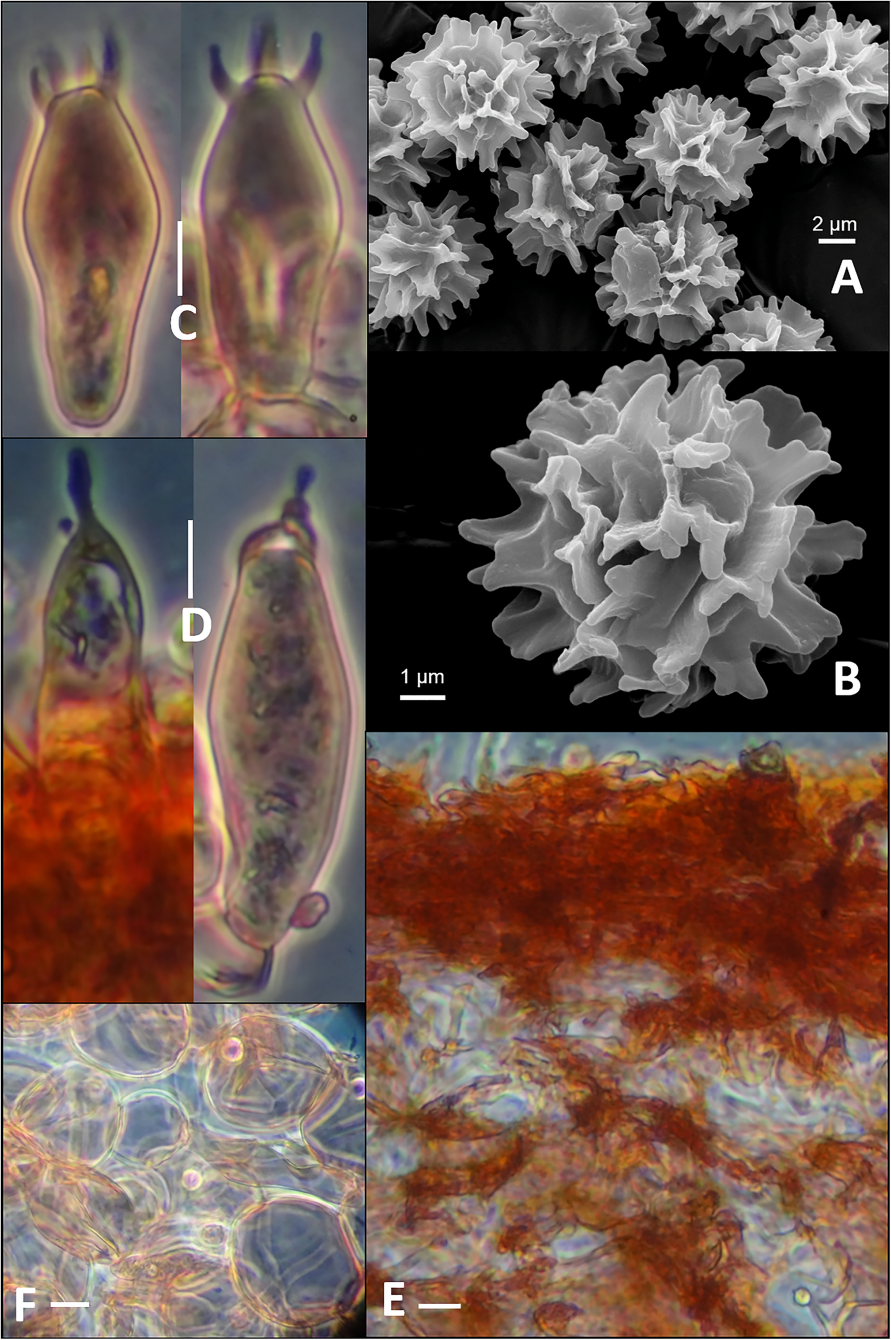
Microscopic features of *Russula alatoreticula* (CAL-1271, holotype). (A-B) Scanning Electron Micrograph of basidiospores. (C) Basidia. (D) Hymenial cystidia (gill sides) as observed in Congo red. (E) Pileipellis. (F) Sphaerocytes. Scale bars: 10 μm in C-F.

Basidiospore globose to sub-globose, (6.5–)6.8–7.0–7.2(–7.5) × 5.4–6.2–6.3(–7.2) μm, Q = 1.05–1.1–1.2, ornamentation composed of very high (1.3–2.1 μm high) ridges with crenulated to irregularly interrupted margin that are aligned or connected to give nearly complete reticulum to reticulate-winged fashion (Fig 2A and 2B), amyloid; suprahilar spot amyloid. Basidia (39–)40–45(–49) × 10.8–11.6(–12.5) μm, 4–spored, clavate to sub-clavate, oil-granule present when viewed with KOH, thin walled; sterigmata 4–5.5 × 1.5–2 μm (Fig 2C). Subhymenium pseudoparenchymatic. Lamellar trama composed of 33–39 × 25–34 μm, isodiametric, hyaline nested sphaerocytes. Hymenial cystidia ca. 60–65(–68) × 10.5–11.5 μm on gill sides (Fig 2D), near gill edge ca. 43–50 × 10–11 μm, clavate to subclavate with capitates to moniliform apex, thin-walled, oil granule present when viewed with KOH. Pileipellis sharply delimited from underlying sphaerocytes of the context, distinctly two layered (Fig 2E and 2F); subpellis (39–)42–54(–57) μm dense, rather gelatinized, composed of horizontally oriented hyphae that are ca. 3.5–5.5 μm wide; suprapellis less gelatinized, (28–)43–46(–50) μm deep, composed of ca. 3.5 μm broad hyphae with pyriform to subulate endings. Incrustations absent. Stipitipellis composed of hyaline, septate hyphal cells. Caulocystidia present, 3–3.5(–4) μm broad, narrow. Clamp-connections absent in all parts.

**Habit and habitat.** solitary, common, growing under *Shorea robusta* C.F. Gaertn. tree in the lateritic regions of West Bengal (Fig 1B and 1C).

**Additional specimen(s) examined.** India, West Bengal, Birbhum district, Shantiniketan, 23°40ʹ57ʹʹ N, 87°40ʹ24ʹʹ E, alt. 67 m, P. Mitra, 21 July 2012, CUH AM112; West Midnapur district, near Khairulachak, 22°27'1.94" N, 87°14'49.32" E, alt. 67 m, 24 July 2012, S. Paloi, CUH AM113; Durgapur, 30 August 2013, P. Mitra, CUH AM 114.

**Fig 3:**
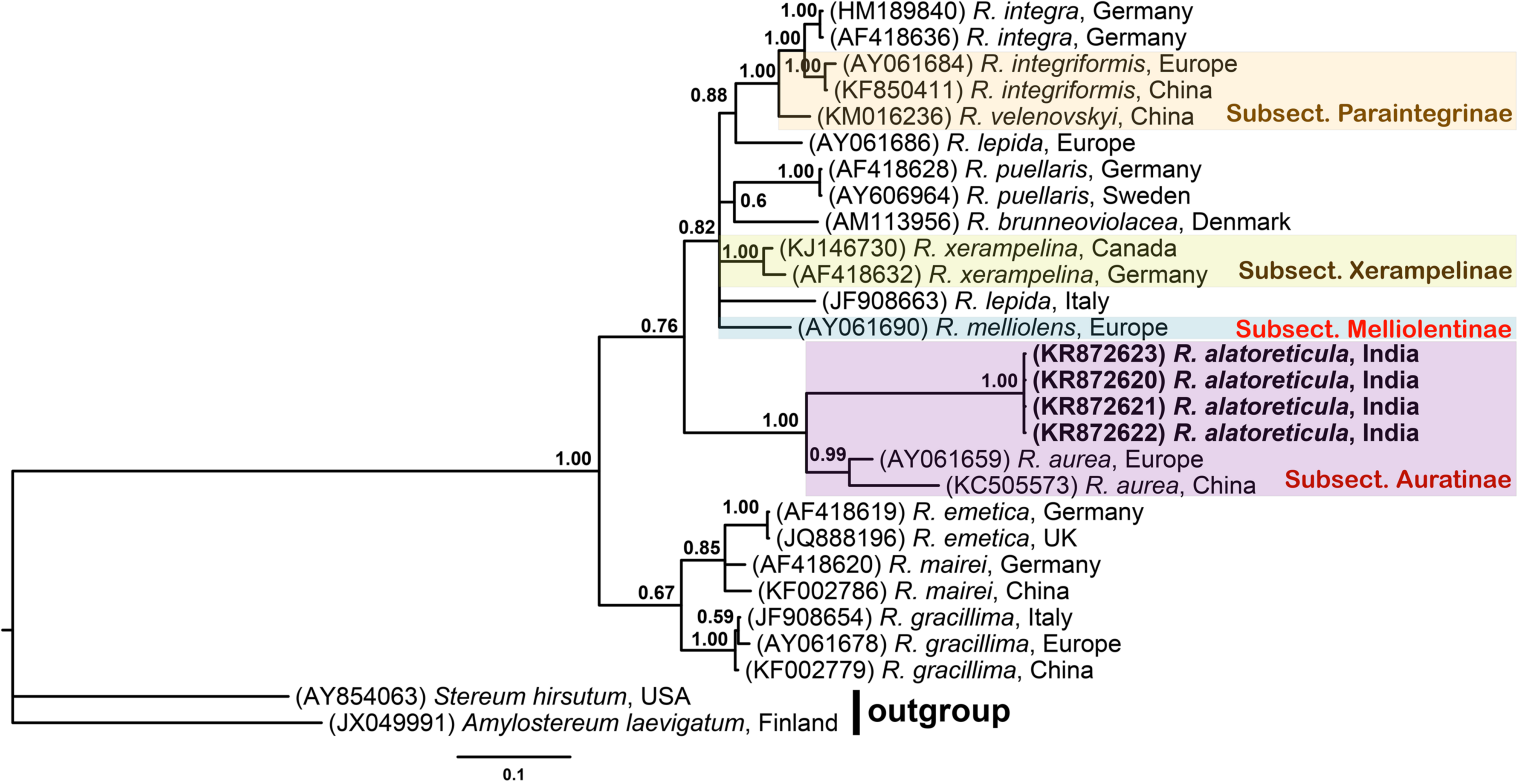
Phylogenetic tree. Consensus phylogram (50% majority rule) resulting from a Bayesian analysis of the nrITS sequence alignment of *Russula* species, showing mean branch lengths, obtained from 10^6^ generations of an MCMC analysis. Bayesian posterior probabilities (≥ 0.50) has been indicated above the branches. The scale bar represents number of expected changes per site. Categorizations of *Russula* species within the phylogenetic tree follows classification of Sarnari [42].

**Note.** Both the macro- and micro-morphological characters (see diagnosis), as well as full support for its phylogenetic placement (PP = 1.00; Fig 3) together with *R*. *aurea*, the type species of the subsect. *Aurantinae*, undoubtedly place the present taxon under the subsect. *Aurantinae*, of sect. *Polychromae*, of the subgen. *Russula* [42].

Within the same subsect. *Aurantinae*, the present species appears close to *Russula aurea* Pers. but, easily distinguish from the later by its smaller size of the basidiospores (6.5–7.5 × 5.4–7.2 μm), very distinct (reticulate-winged up to 2.1 μm high) and unique (in this group) spore ornamentation. In addition, the occurrence of the new species concerned in tropical region and in association with *Shorea robusta* is very striking. In the Indian context, under subgen. *Russula*, among species with more or less same colouration of pileus and smaller size of the basidiospores: *Russula rosea* Pers. differs by the presence of light yellow to pale orange yellow coloured lamellae, pale yellow spore print, and fertile lamellae edge [43,44]; *Russula minutula* var. *robusta* Saini, Atri & Singer has forked lamellae with red coloured margin, and a spore ornamentation that is more typical of subsect. *Roseinae* (partial reticulum or warts connected by veins); *Russula xerampelina* differs by the presence of forked lamellae, white tinged with vinaceous red coloured stipe which turns brown at maturity, ochraceous yellow coloured spore print and basidiospores ornamentation composed of conic to spinoid isolated warts [44]. Among other species belonging to the subgen. *Russula* with white coloured spore print, *R*. *emetica* (Schaeff.) Pers., previously reported to be common in temperate mixed forest of India, differs by its acrid taste, having lamellae forked near the stipe, presence of lamellulae, slightly different size (7.4‒9.8 × 6‒7 μm; Q = 1.14‒1.46) basidiospores, and presence of cylindrical subclavate to clavate pileocystidia [44]; *Russula vaurasiana* K. Das & J.R. Sharma, originally described from India [44], differs by having a pileus coloured deep yellowish pink to reddish orange with light orange yellow tinge towards centre, forked and interveined lamellae, presence of cylindrical to subclavate pileocystidia, and distinctly broader basidiospores (7.3‒9.8 × 6.2‒8 μm).

Among phylogenetically related species: *Russula melliolens* differs by cream coloured spore-print and distinctly broader size basidiospores (9‒11 × 7.7‒9.8 μm) [45]; *R*. *gracillima* Jul. Schäff. has purple to dark vinaceous or violaceous pileus, yellowish white to saffron luteous spore print, cylindrical to clavate pileocystidia, and basidiospores ornamentation composed of mostly conic isolated warts [44]; *Russula velenovskyi* Melzer & Zvára, originally described from Czech Republic and subsequently reported to be common in Europe, differs by its ochraceous spore print, coarser acid-fast taste, obovate basidiospores, and presence of pileocystidia [46]; and *R*. *integriformis* differs by having brownish yellow coloured pileus, sweet taste, yellow spore print, and fusiform hymenial cystidia [47].

### Phylogenetic analysis

Sequencing products of the newly described species ranged from 662 to 673 nucleotides. Based on the nrITS sequences obtained in this study and from GenBank, the phylogenetic relationships of *R*. *alatoreticula* was inferred from MCMC analyses. ITS sequences were aligned and the ends trimmed to create a dataset of 662 base pairs. Bayesian analyses reached a standard deviation of split frequencies of 0.004 after 10^6^ generations. The initial 25% trees recovered were excluded as the burn-in and the remaining trees obtained were then used to estimate the posterior probabilities of the group (Fig 3). Accession numbers of newly generated nrITS sequences, as well as other GenBank sequences used for conducting the phylogenetic analysis, are presented in Fig 3.

### Extraction parameters and physico-chemical characterization of Rusalan

The extraction procedure is considered to have a significant impact on yield and structural characteristics of polysaccharide as well as their biological activities. However, conventional heated reflux extraction is the most common method for isolation of polysaccharide [48]. Therefore, in the present study crude polysaccharide from *R*. *alatoreticula* was prepared using the common method in a modified way. The first step of methodology included an incubation of mushroom powder in ethanol for eliminating unwanted molecules such as lipids, pigments, monosaccharides, oligosaccharides and other small molecular weight components. Afterwards, filtered powder was subjected to hot water extraction, where boiling temperature was adopted with simultaneous shaking for about 6 h. Finally, the water soluble crude polysaccharide, Rusalan, was obtained as a whitish powder following ethanol precipitation with a yield of 3.07% of crude material. The observation was in agreement with previous reports where quantity of harvested polysaccharide from mushrooms have been reported to be in the range of 1.51–5.24% [29,49,50].

To depict the structural characteristics, Rusalan was evaluated using several physico-chemical parameters. As shown in Table 2, the crude polysaccharide was consisted mainly of carbohydrate with small amounts of protein. Besides, glucan was detected as the main constituents where glucose linked by β-glycosidic linkages were detected as the predominant form. According to previous literature, the chemical composition of Rusalan was found to be better than *Macrocybe gigantea* [29], *Pleurotus florida* [51], *Ganoderma applanatum* [52] and closely related to *Termitomyces eurhizus* [49]. Consequently, *R*. *alatoreticula* could be regarded as a good source of crude polysaccharide enriched with β-glucan.

**Table 2:** Chemical characterization of crude polysaccharide, Rusalan from *Russula alatoreticula*, (mean ± standard deviation; n = 3).

Characters	Rusalan
Yield of polysaccharide gm/ 100 gm of dry powder	3.07 ± 0.158
Total carbohydrate gm/ 100 gm of polysaccharide	37.33 ± 4.17
Total protein gm/ 100 gm of polysaccharide	5.5 ± 0.1
Total glucan gm/ 100 gm of polysaccharide	23.86 ± 2.9
α glucan gm/ 100 gm of polysaccharide	7.13 ± 0.07
β glucan gm/ 100 gm of polysaccharide	16.73 ± 2.8
Helical conformation	Triple helix
Monosaccharide composition	Mannose: Glucose: Galactose = 1: 6.06: 2.38

#### Depiction of helical conformation by congo red reaction

**Fig 4:**
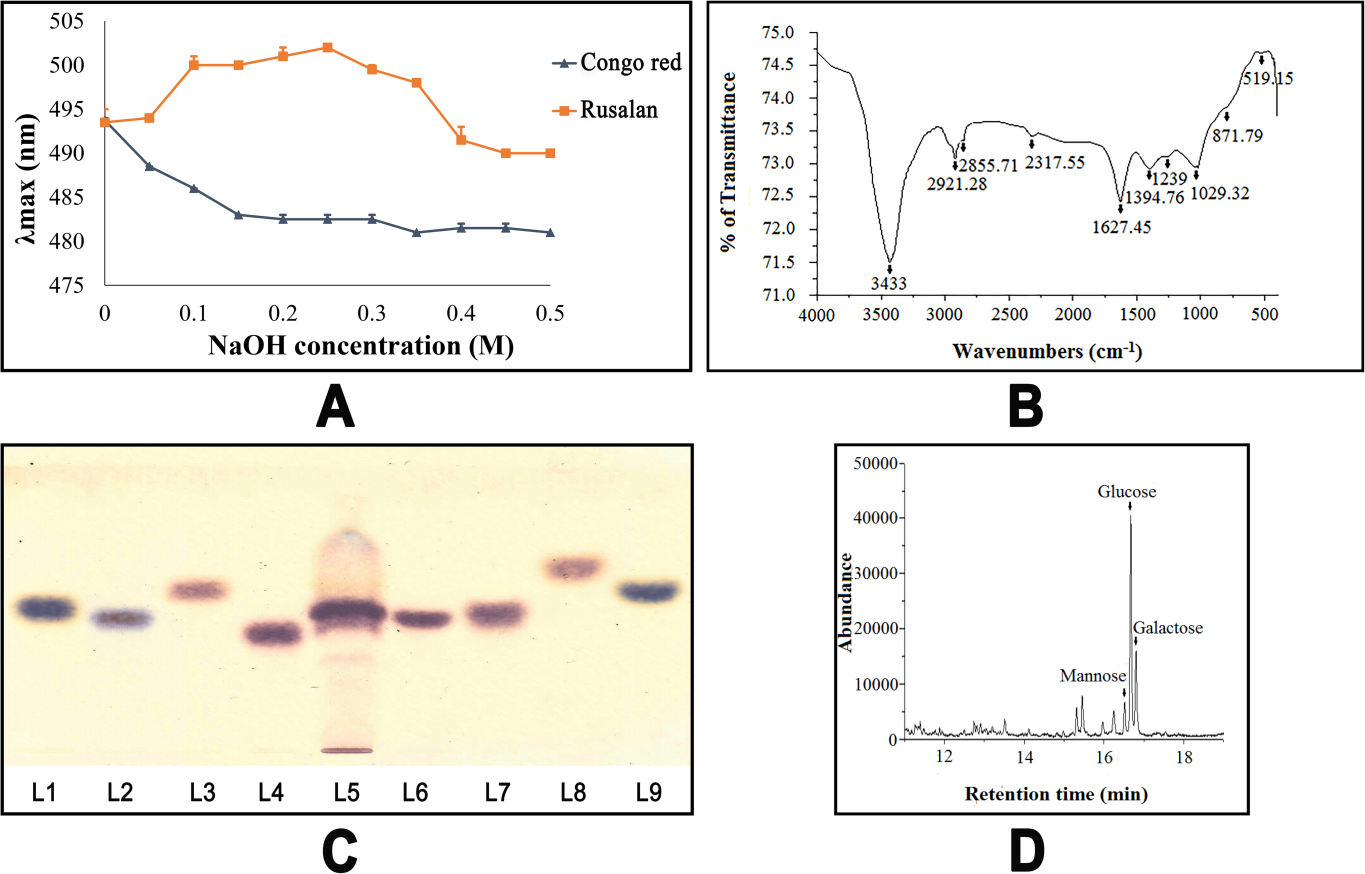
Structural and molecular characterization of crude polysaccharide, Rusalan, isolated from *Russula alatoreticula*. (A) Changes in absorption maximum of Congo red-polysaccharide complex at various concentrations of sodium hydroxide solution (B) FT-IR spectra (C) Identification of monosaccharides in hydrolysed polysaccharides by HPTLC. Lanes: 1: L-arabinose, 2: D-fructose, 3: D-fucose, 4: D-galactose, 5: Rusalan, 6: D-glucose, 7: D-mannose, 8: D-rhamnose, 9: D-xylose (D) GC-MS chromatogram of derivatized Rusalan (Retention time of D-mannose: 16.6 min, D-glucose: 16.7 min, D-galactose: 16.8 min).

Glucans with high degrees of β (1→6) or (1→3) glycosidic bonds exist generally in triple helical conformation and can form complexes with Congo red in dilute alkaline solution. The complex formation is usually characterized by means of shift in visible absorption maxima (λmax) of Congo red spectrum. However, a strong alkaline environment can break the helical structure into a disordered form, as a result λmax changes [53]. As shown in Fig 4A, λmax of the mixture of Congo red and Rusalan presented a shift of 9 nm (from 493 to 502 nm) in 0.1 M NaOH solution and further it decreased with increment of alkali concentration. Therefore, the result suggested that Rusalan might had triple helical conformation which lost at higher concentrations of 0.3 M NaOH. This ordered helical structure has been recently reported as the most stable form of polysaccharide. At present, all the major therapeutic mushroom polysaccharides available in market such as schizophyllan, lentinan and scleroglucan are known to possess that specific construction [54].

#### FT-IR of Rusalan

FT-IR spectroscopy was performed herein to identify structure of polymers present in Rusalan and the infrared spectra confirmed existence of functional groups corresponding to carbohydrate (Fig 4B). Presence of broad and strong absorption band at 3433 cm^−1^ was due to stretching vibrations of hydroxyl groups. Peak at 2921.28 cm^−1^ corresponded to symmetric stretching vibrations of CH group. The signal of amide band at 1627.45 cm^−1^ indicated presence of some residual protein in crude polysaccharide. Band at 1394.76 cm^−1^ resembled to OH group of phenolic compounds. Absorption peaks between 1250 and 950 cm^-1^ (i.e. 1239.63 cm^-1^, 1029.32 cm^-1^) indicated that sugar rings were of pyranose forms. The characteristic peak at 871.79 cm^−1^ denoted existence of β-glycosidic bonds. Band at 720.8 cm^−1^ indicated presence of mannose in Rusalan. The band visible in between 500 and 749 cm^-1^ (i.e. 519.15 cm^-1^) signified presence of R–CH group [55–58]. Overall, the spectra showed high absorbance at wavenumbers characteristic of polysaccharide: 3400 cm^−1^ OH and 1200–800 cm^−1^ carbohydrate. Based on aforementioned results, it can be concluded that Rusalan was composed of sugar units in β-configuration and pyranose rings. Small amount of lipid and protein were also extracted together with polysaccharides.

#### Determination of monosaccharide composition

It is well known that the biological properties of carbohydrate are greatly influenced by monosaccharide ratio that forms the backbone of polysaccharides. For preliminary characterization of the monomer types present in Rusalan, HPTLC was performed followed by orcinol-H_2_SO_4_ spray. The reagent reacts differently to each monosaccharide showing a specific colour and colour intensities vary depending on sugar concentration [59]. In the present study, the chromatogram of Rusalan demonstrated to be consisted of three monosaccharides i.e. galactose, glucose and mannose while, other six monomers were not detected (Fig 4C). The result was further confirmed by using GC coupled with MS and the approach required derivatization of Rusalan by hydrolysis, reduction followed by acetylation. The fingerprint profile was detected as similar to previous result as identified by HPTLC (Fig 4D).

### Evaluation of antioxidant activities of Rusalan *in vitro*

**Fig 5:**
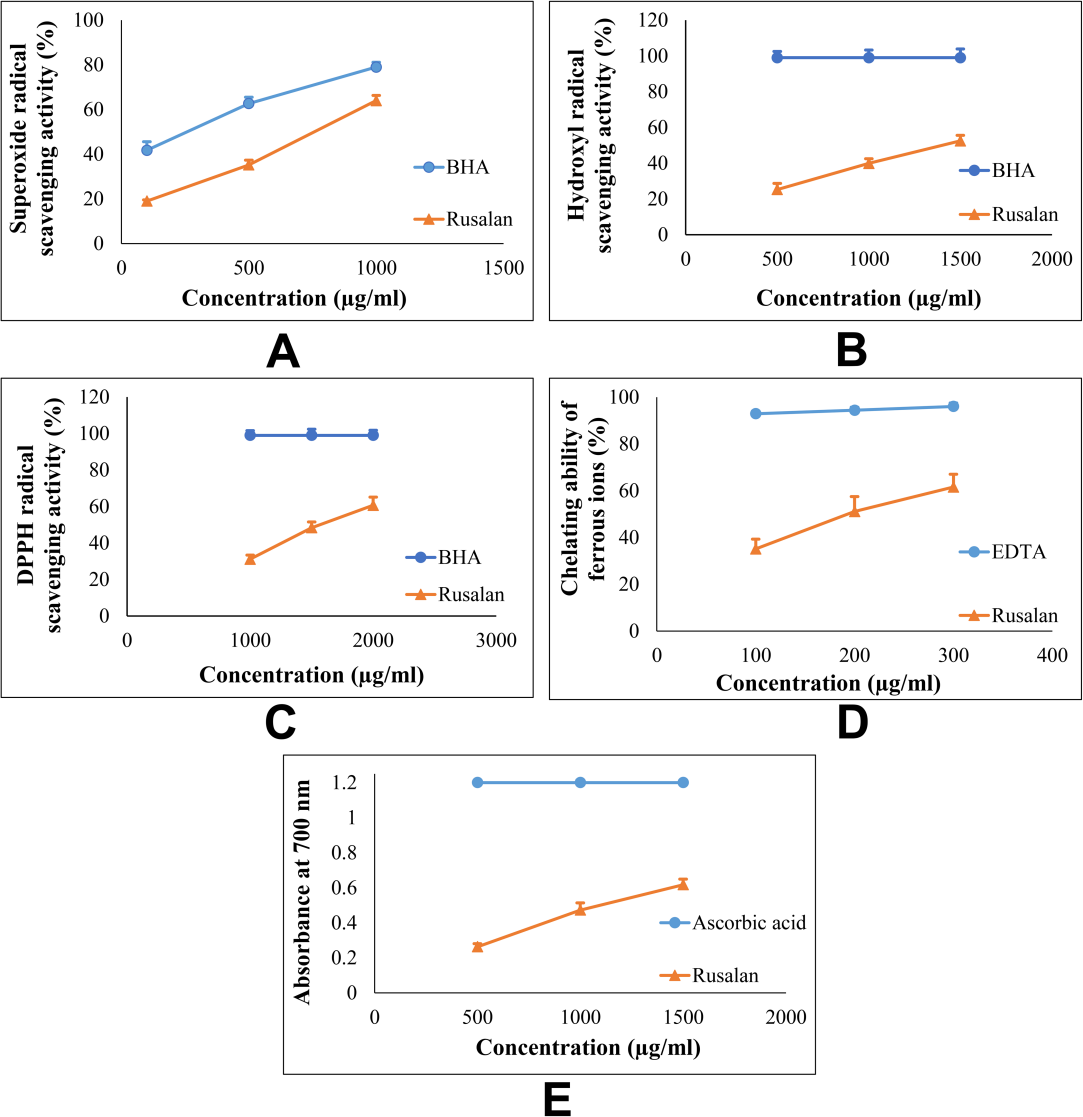
Antioxidant activity of crude polysaccharide, Rusalan, prepared from *Russula alatoreticula*. (A) Superoxide radical scavenging activity (B) Hydroxyl radical scavenging activity (C) DPPH radical scavenging activity (D) Chelating ability of ferrous ion (E) Reducing power. Results were represented as mean ± standard deviation of triplicate experiments.

The antioxidant activity of a component is dependent on its variable characteristics such as scavenging or quenching free radicals, chelation of transition metals, inhibition of lipid peroxidation and reduction power [59]. Thus, it would be difficult to predict the potentiality of antioxidants by performing a single assay. In this circumference, six different *in vitro* techniques were executed in the present study to evaluate antioxidant ability of Rusalan and the effects have been presented in Table 3. For estimation of radical scavenging activity three methods were performed such as superoxide, hydroxyl and DPPH radical scavenging assays. For the first process, riboflavin-light-NBT method was followed based on generation of blue coloured formazan by action of superoxide radicals. Presence of antioxidants prevented formazan formation and as a result colour intensity decreased. Results demonstrated that Rusalan exhibited strong quenching effects of the radicals which increased dose-dependently. As presented in Fig 5A, Rusalan was able to inhibit 19.05% and 35.23% radicals at concentrations of 100 and 500 μg/ml respectively which reached up to 63.98% at the dose of 1000 μg/ml. Based on the outcome, it can be postulated that Rusalan may possess stronger potentiality than crude and pure polysaccharides of *Russula virescens* [60]. Besides, another approach was adopted to determine radical scavenging properties of the extract. In this case, hydroxyl radicals were generated using Fenton’s reaction that gradually produced MDA-TBA pink chromogen and presence of antioxidants scavenged radicals causing inhibition of the whole process. Results of the assay showed that Rusalan was able to scavenge the radicals at relatively low concentrations in a dose dependent manner (Fig 5B). The quenching activities of Rusalan were found to be 25.29%, 39.88%, and 52.5% at the concentrations of 500, 1000 and 1500 μg/ml respectively. On comparison to previous publications, the extract exhibited better radical scavenging potentiality than that of crude polysaccharides extracted from *Auricularia auricula*, *Agaricus bisporus*, *Lentinus edodes* and *Flammulina velutipes* [61]. DPPH^.^, a commercially available free radical, was evaluated against the formulation for better visualization of radical scavenging activity. Results indicated that at 1000 μg/ml concentration, Rusalan exhibited strong quenching potentiality (31.09%) that gradually increased to 48.37% and 60.67% at the doses of 1500 and 2000 μg/ml respectively (Fig 5C). However, the potentiality was found to be less effective than crude polysaccharide extracted from *M*. *gigantea* [29]. Further, metal ion binding capacity of the fraction was estimated following method of chelating ability of ferrous ion. The assay was based on formation of ferrous ion-ferrozine complex which became interrupted in presence of antioxidative component. In this technique, the extract demonstrated marked Fe^2+^ binding ability at relatively low concentrations that incremented with increase of concentrations (Fig 5D). At the level of 100, 200 and 300 μg/ml, the formulation was able to chelate 35.1%, 51.09% and 61.55% of ferrous ions respectively. Recently in a previous study, antioxidant activity of partially purified crude polysaccharide extract from the mushroom, *Schizophyllum commune*, have been reported which appeared to be much poorer than Rusalan [62]. Further, ferricyanide/prussian blue and total antioxidant assay by phosphomolybdenum methods were carried out to evaluate the reducing power of Rusalan. The previous assay was based on electron-donating ability of antioxidants to reduce yellow coloured ferric to blue coloured ferrous form. According to results, the crude polysaccharide exhibited strong reduction power which incremented dose dependently (Fig 5E). At the concentration of 500 and 1000 μg/ml the reducing power have been detected as 0.26 and 0.47 that gradually incremented to 0.62 at the level of 1500 μg/ml. However, the sample exhibited antioxidant activities less effective than a purified β-glucan of *Russula albonigra* [63]. Finally, total antioxidant capacity assay was performed based on reduction of Mo(VI) to Mo(V) in presence of antioxidant. Results indicated that 1000 μg of Rusalan acted as the equivalent to 4.9 μg of ascorbic acid. Thus, based on the results of all aforementioned assays, it could be stated that Rusalan may possess comparatively stronger antioxidant ability.

**Table 3:** Antioxidant activity of crude polysaccharide, Rusalan, isolated from *Russula alatoreticula*. The results are presented in EC_50_ values (mean ± standard deviation; n = 3) corresponding to 50% of antioxidant activity or 0.5 of absorbance for reducing power assay. BHA was used as standard in superoxide, hydroxyl and DPPH radical scavenging methods. Ascorbic acid was considered as standard in reducing power and total antioxidant capacity assays, while EDTA was adopted as a positive control in chelating ability of ferrous ion method. In each row, different letters mean significant differences between sample and standard (*p* < 0.05).

Antioxidant parameters		Rusalan	Standard
EC_50_ value (μg/ml)	Scavenging ability of superoxide radical	742 ± 25 ^a^	261 ± 36^b^
	Scavenging ability of hydroxyl radical	1305 ± 90 ^a^	69 ± 1^b^
	Scavenging ability of DPPH radical	1328 ± 58 ^a^	2.15 ± 0.25^b^
	Reducing power	1237 ± 76 ^a^	14.5 ± 5^b^
	Chelating ability of ferrous ion	190 ± 10 ^a^	2.54 ± 0.5^b^
Total antioxidant activity by phosphomolybdenum method (μg ascorbic acid equivalent/mg of dry polysaccharide)		4.9 ± 0.9	-

### Estimation of antibacterial potentiality of Rusalan

Antibacterial activity of Rusalan was assessed against six different bacteria, some of which are involved in diseases related to respiratory and gastrointestinal tracts. Results indicated that crude polysaccharide was the most effective in inhibiting growth of *S*. *aureus*; while *B*. *subtilis* was also detected as sensitive to the fraction. In contrast, Rusalan did not show any activity against rest of the organisms especially Gram negative bacteria even when higher concentration was applied (Table 4). According to previous literature, Gram positive bacteria such as *S*. *aureus* has revealed high susceptibility to mushroom extracts. While, Gram negative bacteria are difficult to be inhibited due to their cell wall structure [64]. However, our findings indicated that Rusalan was more active in inhibition of bacterial growth than methanol fraction of *Russula delica* [65].

**Table 4:** Antibacterial activity of crude polysaccharide, Rusalan, isolated from *Russula alatoreticula* as determined by minimum inhibitory concentration (MIC) value (mean ± standard deviation; n = 3).

Type of bacteria	Name of bacteria	Rusalan (mg/ml)	Streptomycin (μg/ml)
Gram positive	*Listeria monocytogenes*	>10	4.68 ± 0.17
	*Staphylococcus aureus*	1.029 ± 0.161	6.295 ± 0.164
	*Bacillus subtilis*	1.131 ± 0.257	5.608 ± 0.009
Gram negative	*Escherichia coli*	>10	5.415 ± 0.11
	*Salmonella typhimurium*	>10	5.092 ± 0.028
	*Klebsiella pneumoniae*	>10	5.294 ± 0.143

### Determination of immuno-modulatory activity of Rusalan

#### Effect on Raw 264.7 cell proliferation

**Fig 6:**
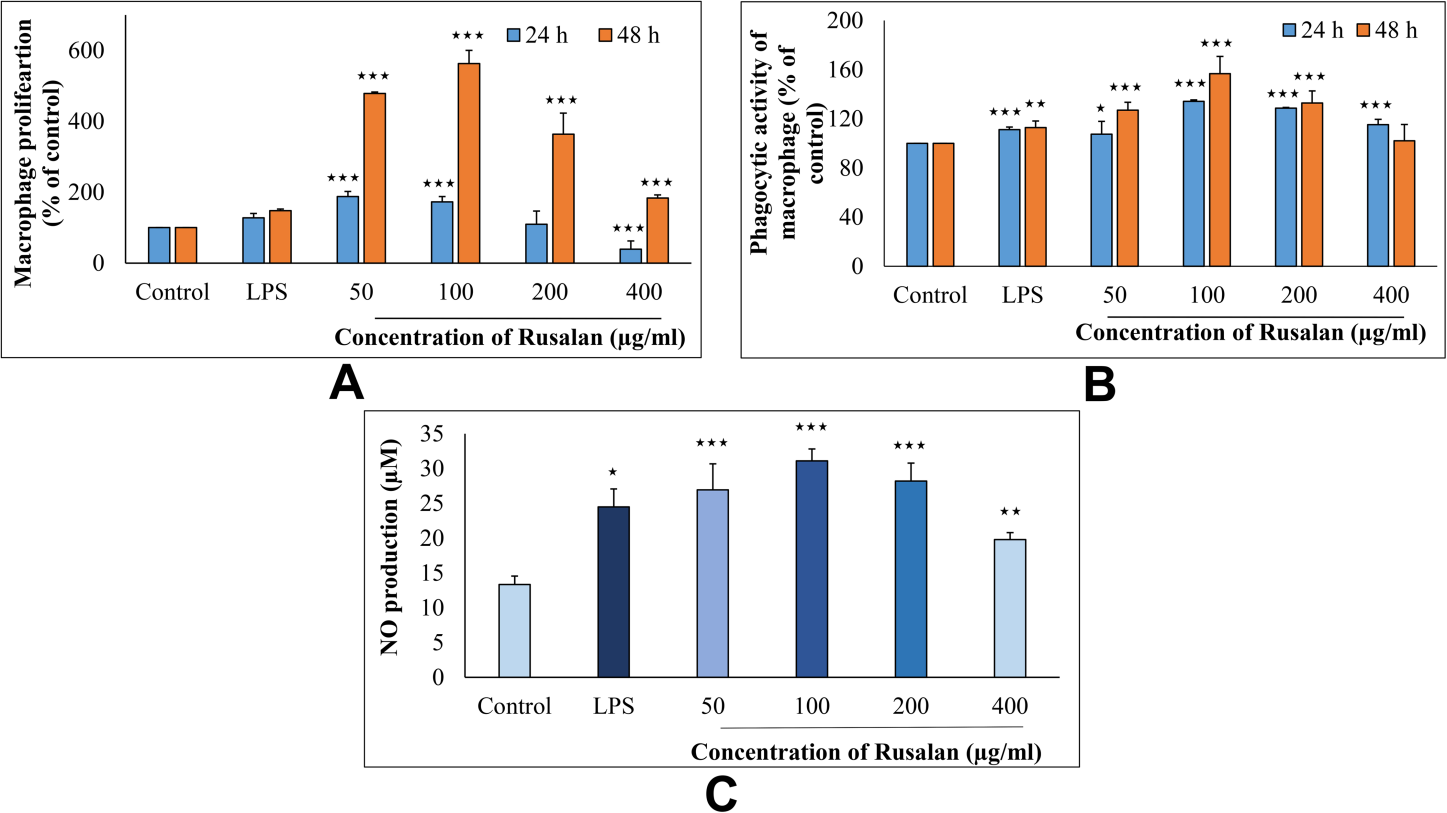
Effect of crude polysaccharide, Rusalan, from *Russula alatoreticula* on activity of macrophages. (A) Proliferation was monitored in treatment of Rusalan for 24 and 48 h by WST method and expressed in relation (%) to negative control. (B) Phagocytosis in relation (%) to control was determined by neutral red method. (C) Release of nitric oxide in cell supernatant was quantified using Griess reagent. In all assays LPS at the concentration of 5 μg/ml was used as positive control. Values were presented as mean ± standard deviation of at least three independent experiments. (*p,0.05, **p,0.01, ***p,0.001, unpaired t-test).

Activation of macrophages is an important step in defence mechanism system to protect the host from pathogenic infection. Thus, at first influence of Rusalan on macrophage cellular viability was examined using WST reagent which produces orange coloured formazan dye in direct proportional to number of living cells. As shown in Fig 6A, proliferation of cells was enhanced significantly at all concentrations of the crude polysaccharide applied. It has been estimated that after treatment of polysaccharides (50, 100, 200 and 400 μg/ml) for 24 h, proliferation of macrophage cells increased to 187.65%, 172.85%, 109.36% and 39.08% respectively in comparison to negative control. While after 48 h, the viability elevated to 478.6%, 562.68%, 363.5% and 183.32% at those above mentioned concentrations. Data also suggested that the extract might activate macrophages more efficiently than the positive control because LPS incremented proliferation up to 127.57% and 147.74% after 24 and 48 h of treatment respectively. However, the results allowed to conclude that Rusalan presented a non-toxic safety profile to murine macrophages over the entire concentration range evaluated.

#### Effect on phagocytic activity of macrophage cells

To eliminate foreign matters, macrophages have ability to phagocytize the pathogens that plays an important role in host defence. Thus, phagocytosis is considered as an indication of macrophage activity and it represents the final essential step of immunological defence system [4]. In the present assay, influence of Rusalan on phagocytic activity of macrophages was determined by measuring the amount of neutral red internalized in cells. Results clearly indicated that crude polysaccharide promoted phagocytosis of macrophages in a dose and time dependent manner (Fig 6B). After 24 h of treatment, the activity was increased by 107.41%, 134.17%, 128.72% and 115.25% due to treatment of 50, 100, 200 and 400 μg/ml of Rusalan while after 48 h, the activity was 126.93%, 156.72%, 132.86% and 102.02% in comparison with negative control. On the other hand, LPS induced 109.76% and 116.67% after treatment of 24 and 48 h respectively. Thus, it could be predicted that the extract could induce innate immune response by regulating phagocytosis of macrophage.

#### Effect on NO production in macrophages

Production of NO is another killing mechanism of an activated macrophage that works in combination with hydrogen peroxide or superoxides. The reaction leads to formation of peroxynitrite radicals that kill phagocytosed microbes inside macrophages. Thus, generation of NO can be used as an indication for activation of macrophages [66]. In the present assay, nitrite concentrations in the supernatant of stimulated macrophages were determined by Griess reagent as a reflection of NO production. As shown in Fig 6C, Rusalan had a strong potential on stimulating NO production in macrophages in a concentration dependent trend. In case of control and LPS stimulated sets 13.33 and 24.47 μM NO was quantified; while the crude polysaccharide induced 26.92, 31.08, 28.2 and 19.77 μM NO production in RAW 264.7 cells at the level of 50, 100, 200 and 400 μg/ml respectively.

#### Detection of morphological changes of macrophages

**Fig 7:**
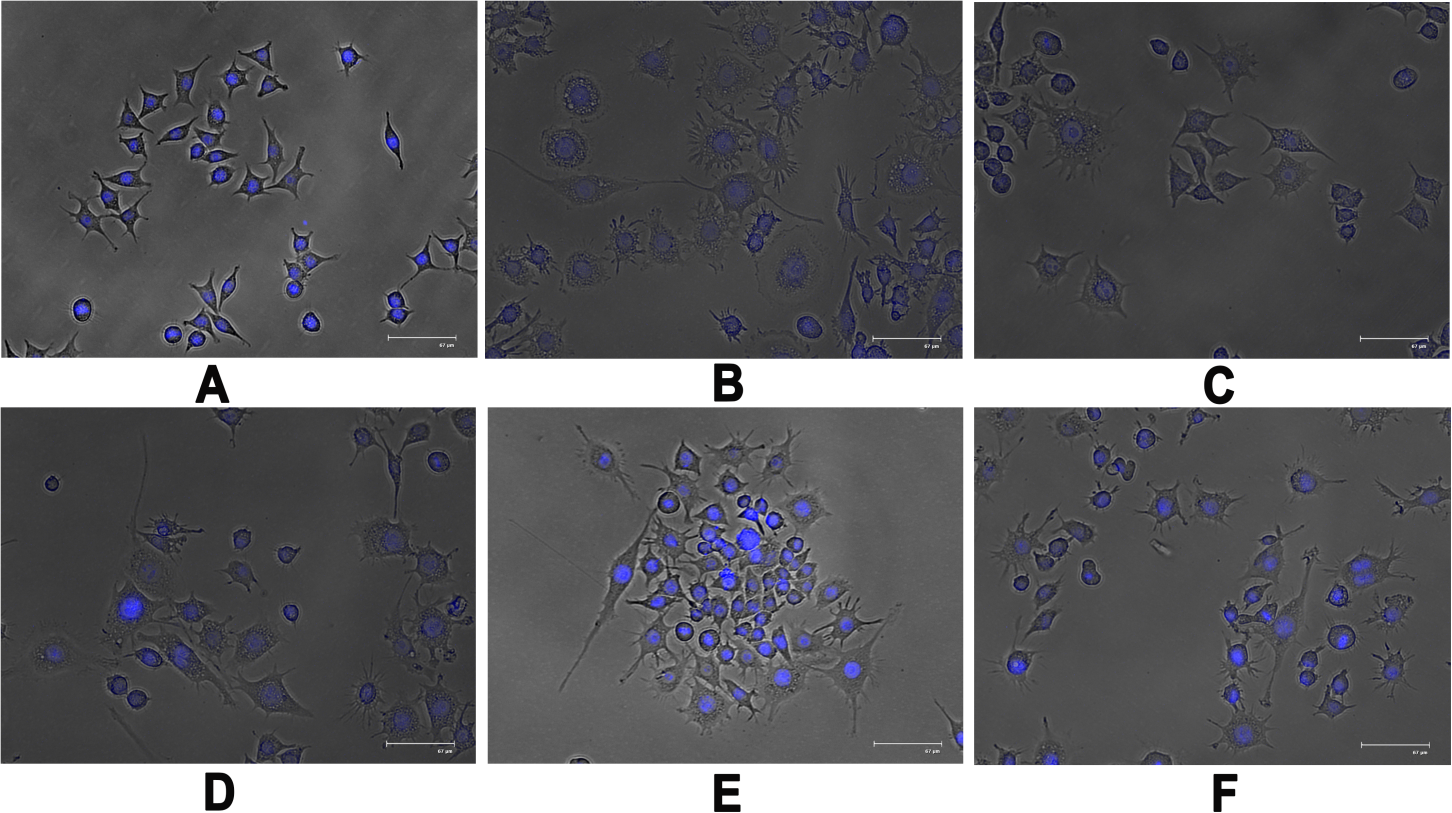
Effect of crude polysaccharide, Rusalan, isolated from *Russula alatoreticula* on morphology of macrophages. Cells were incubated for 24 h with different concentrations of Rusalan where LPS at 5 μg/ml concentration was used as a positive control. Afterwards, cells were fixed, stained with DAPI, subjected to fluorescence microscopy and images were captured. (A) Negative control (B) LPS (C) 50 μg/ml (D) 100 μg/ml (E) 200 μg/ml (F) 400 μg/ml.

When macrophages encounter a stimulus, they are known to undergo certain morphodynamics such as increase in cell size, production of thin sheets of cell edges called filopodia or lamellopodia [67,68]. Recently, it has been evidenced that NF-κB plays an important role in regulation of the formation of lamellipodia and induction of morphological changes [67]. Consequently, to investigate effects of Rusalan on macrophage cell morphology, Raw 264.7 cells were analysed in presence of crude polysaccharide at different concentrations for 24 h. LPS was used a standard as it induces structural changes that are definite sign of macrophage activation. Microscopic analysis showed that after LPS stimulation, a large number of thin sheet extensions were produced from surface of macrophage along with increased spreading of cells. Rusalan treatment also induced similar kind of morphological changes in all examined concentrations, in contrast to unstimulated cells that did not show any morphological alterations (Fig 7).

#### Effect on production of intracellular ROS

**Fig 8:**
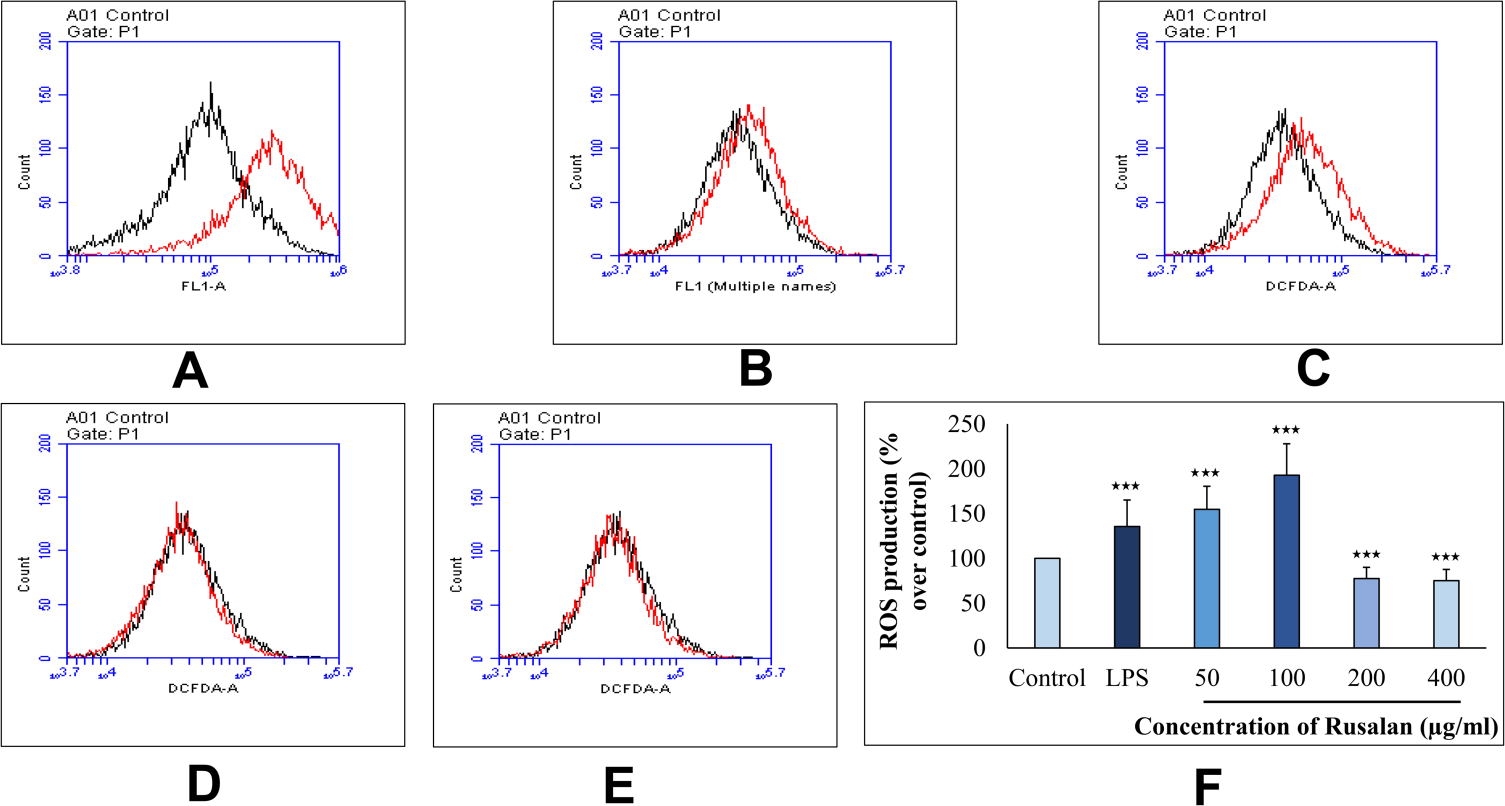
Effect of crude polysaccharide, Rusalan, isolated from *Russula alatoreticula* on intracellular production of ROS in macrophages. Raw 264.7 cells were treated with Rusalan or LPS and after 24 h intracellular ROS generation was determined by flow cytometry using DCFDA dye. Red coloured graphs represent log fluorescence intensity of oxidative product of DCFDA treated with (A) LPS at 5 μg/ml concentration and Rusalan at variable doses such as (B) 50 μg/ml (C) 100 μg/ml (D) 200 μg/ml (E) 400 μg/ml concentration in comparison with negative control denoted by black coloured graph. (F) Relative fluorescence intensity was also analysed in detail. Data were represented as mean ± standard deviation of three independent experiments. (*p,0.05, **p,0.01, ***p,0.001, unpaired t-test).

In addition to phagocytosis, generation of ROS inside phagocytes also plays a key role in the process of bacterial killing as it signals for cytokine production via activating NF-κB pathway. In fact, it has been proved that persons with lacking ability of ROS production suffer from chronic granulomatous disease as they are inefficient to clear pathogens properly. Thus, enhanced ROS formation may be essential for host survival against microbial and fungal pathogens [69]. Result of the present study showed that Rusalan was capable of inducing ROS production inside macrophages as measured by fluorescent oxidative product of DCFDH. In case of 50, 100, 200 and 400 μg/ml treated cells, the fluorescence intensity increased to 154.82%, 192.9%, 77.52% and 75.26% respectively in comparison with control. While the standard at the concentration of 5 μg/ml could enhance the oxidative bursts only by 135.722% in RAW 264.7 cells (Fig 8).

#### Measurement of gene expression by Reverse Transcriptase-PCR analysis

Cytokines and chemokines are considered as potent signalling molecules produced by macrophages which connect both innate and adaptive immunity. However, secretion of them requires activation of intracellular signalling cascades such as MAPK and NF-κB pathways that regulate expression of iNOS, TNF-α and IL-6 genes [70]. Among them, TNF-α has been noticed as the first cytokine to be secreted in response to pathogens that in turn activates the macrophages itself. Gradually, stimulated macrophages produce other effectors such as IL-6 which is important for differentiation of B cells into plasma cells and activation of cytotoxic T cells [4]. In addition, macrophages express iNOS that signals for production of NO encouraging a highly microbicidal environment [69]. The overproduction of NO further stimulates synthesis of COX-2 that functions in conversion of arachidonic acid to prostaglandins [71, 72].

**Fig 9:**
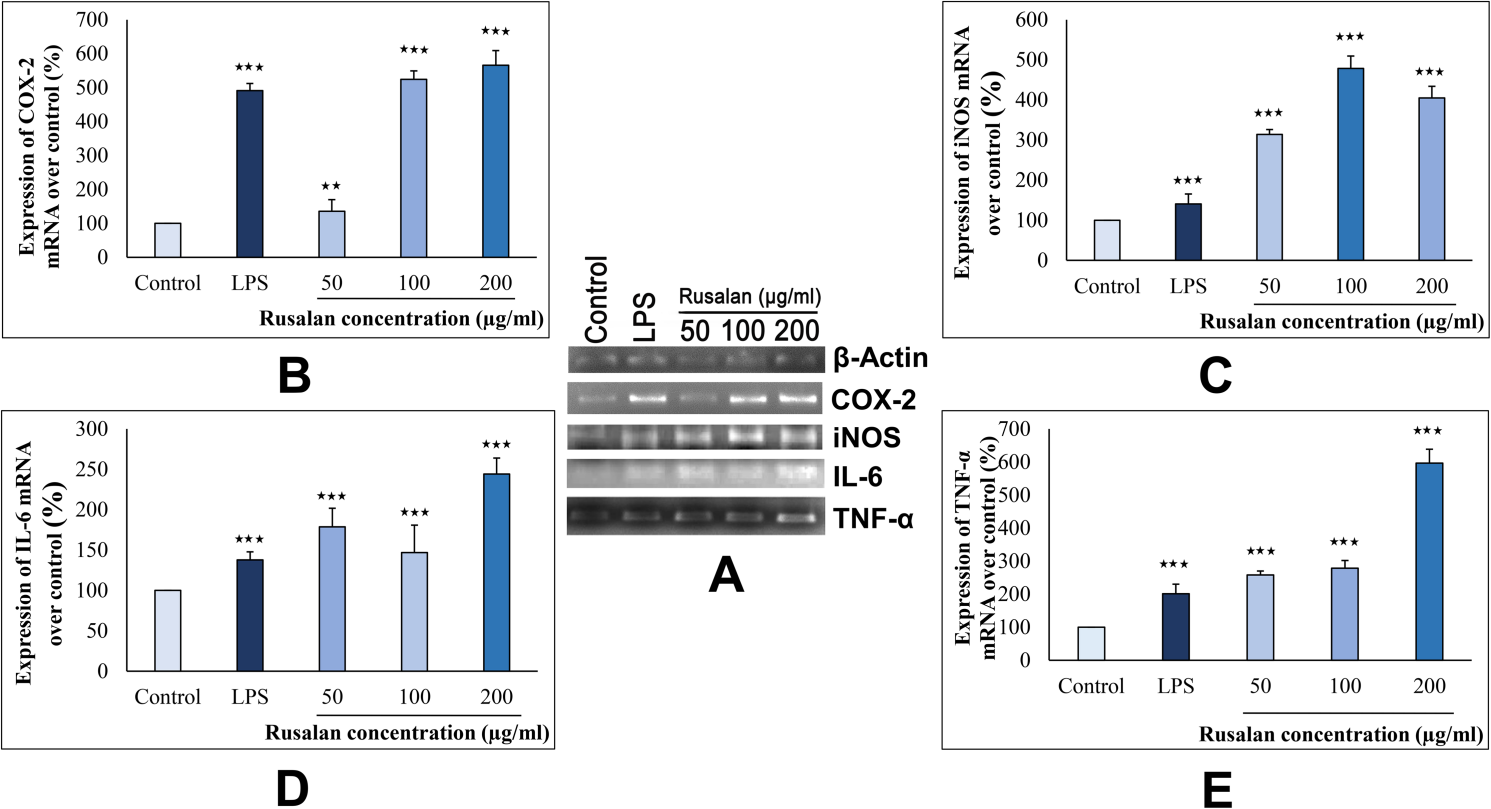
Analysis of mechanism of action by crude polysaccharide, Rusalan, isolated from *Russula alatoreticula* in Raw 264.7 cells. (A) Total RNA was isolated from macrophage cells after 24 h incubation either with LPS (5 μg/ml concentration) or Rusalan (50, 100 and 200 μg/ml concentration) along with untreated cells. cDNA was prepared from respective RNA samples and semi-quantitative reverse transcriptase PCR was performed to analyse the expression of four different genes where β-Actin was considered as a house keeping gene. Further, the band intensities were quantified by ImageJ software to signify increase in transcription level of corresponding genes in relation (%) to control: (B) COX-2, (C) iNOS, (D) IL-6 (E) TNF-α. Values were represented as mean ± standard deviation of two independent experiments. (*p,0.05, **p,0.01, ***p,0.001, unpaired t-test).

In order to investigate the mechanism of action of Rusalan towards stimulation of macrophages, the crude polysaccharide was incubated with Raw 264.7 cells for 24 h. In this experiment, concentration of 50, 100 and 200 μg/ml of crude polysaccharide were selected as the highest dose as (400 μg/ml) could not activate macrophages effectively according to previous observations. As shown in Fig 9, the levels of all four genes were visibly increased in treatment of Rusalan in contrast to negative control and LPS. Analysis by ImageJ software suggested that Rusalan at 100 μg/ml concentration increased the mRNA levels of iNOS, TNF-α, COX-2 and IL-6 by 478.3%, 278.72%, 524,12% and 146.89% respectively over control. Conversely, LPS upregulated these genes only by 140.48%, 201.15%, 491.38% and 137.85% indicating strong promoting effect of Rusalan. So, the results demonstrated that these genes are definitely involved in Rusalan mediated immune-stimulatory activities in murine macrophage cells.

## Conclusions

The present investigation provided information to confirm novelty of the studied mushroom, *R*. *alatoreticula*, based on morphological characteristics and molecular phylogenetic study using ITS region. In addition, the work also demonstrated that crude polysaccharide extracted from the macrofungus, Rusalan, as a potent therapeutic agent with several therapeutic effects. Evaluation of antioxidant activity indicated that the fraction exhibited significant potentiality in hydroxyl, superoxide and DPPH radicals scavenging activity, chelating ability of metal ions along with donating electron. Besides, the extract also revealed to be capable of inhibiting growth of certain pathogenic bacteria. Nevertheless, the crude polysaccharide showed high immune-modulatory activity without cytotoxicity in terms of stimulation of phagocytic activity, production of NO by activating iNOS gene expression, generation of intracellular ROS and upregulation of transcription level of cytokines in macrophage Raw 264.7 cells. Interestingly, the fraction was determined to be mainly consisted of carbohydrate in triple helical conformation where glucose (especially β-glucan), galactose and mannose were the main constituents. Based on the outcome of the present study, Rusalan could be regarded as a novel ingredient for pharmaceutical use against free radical, antibiotic resistant pathogens and hypo-immunity. Further studies on isolation of active component and its relation with bioactivity are presently in progress.
